# Reporting transparency: making the ethical mandate explicit

**DOI:** 10.1186/s12916-016-0587-5

**Published:** 2016-03-16

**Authors:** Stuart G. Nicholls, Sinéad M. Langan, Eric I. Benchimol, David Moher

**Affiliations:** Children’s Hospital of Eastern Ontario (CHEO) Research Institute, c/o RI Administration Offices, Research Building 1, 401 Smyth Road, Ottawa, ON K1H 8L1 Canada; School of Epidemiology, Public Health and Preventive Medicine, Faculty of Medicine, University of Ottawa, Ottawa, Canada; Faculty of Epidemiology and Population Health, London School of Hygiene and Tropical Medicine, London, UK; Institute for Clinical Evaluative Sciences, Toronto, Canada; Department of Pediatrics, University of Ottawa, Ottawa, Canada; Ottawa Hospital Research Institute, Ottawa, Canada

**Keywords:** Moral obligations, Publication bias, Research personnel/ethics, Research waste, Social values, Standards

## Abstract

Improving the transparency and quality of reporting in biomedical research is considered ethically important; yet, this is often based on practical reasons such as the facilitation of peer review. Surprisingly, there has been little explicit discussion regarding the ethical obligations that underpin reporting guidelines. In this commentary, we suggest a number of ethical drivers for the improved reporting of research. These ethical drivers relate to researcher integrity as well as to the benefits derived from improved reporting such as the fair use of resources, minimizing risk of harms, and maximizing benefits. Despite their undoubted benefit to reporting completeness, questions remain regarding the extent to which reporting guidelines can influence processes beyond publication, including researcher integrity or the uptake of scientific research findings into policy or practice. Thus, we consider investigation on the effects of reporting guidelines an important step in providing evidence of their benefits.

## Background

Poor research reporting has been estimated to lead to billions of dollars of waste due to unusable results [1]. Key codes of medical practice, such as the Declaration of Helsinki, state that researchers are accountable for the completeness and accuracy of their reports and that these should adhere to accepted guidelines for ethical reporting [2]. Nevertheless, despite such declarations, there has been little explication as to the principles guiding this ethical obligation. The present commentary addresses why researchers should improve their reporting practices, arguing that the important ethical drivers of these obligations are based on the integrity of individuals as researchers as well as the implications for research use, and concluding that reporting guidelines may help us meet these obligations. However, to date, evidence on the latter is lacking.

### Researchers, research integrity, and trustworthiness

From an individual’s integrity perspective, obligations derive from values pertaining to those conducting and reporting research. The onus is placed on individuals being honest in their research, with evidence provided in the form of complete and accurate reporting [3]. Furthermore, the principles of fairness and reciprocity require researchers to contribute to the accumulating pool of scientific knowledge [4]. Appropriate contribution requires the content of publications to be useable. Improved reporting increases the potential for appropriate review and evaluation of research, allowing researchers to meet their obligations by usefully contributing to the collective knowledge upon which they draw.

Improving reporting of research may also allow researchers to fulfil what Carter et al. [4] called the ‘social license’ of research, which requires researchers to go beyond mere compliance with legal mandates and act in accordance with the expectations of society regarding the conduct of research activities, the implication being that poorly reported research fails to meet the societal expectations required for the maintenance of such a license.

### Extrinsic values and implications for research

Obligations regarding reporting also stem from principles external to the integrity of individual researchers and reflect concerns regarding the final use of data and results. These obligations include principles of avoiding harms and promoting benefits, and fair use of resources.

If poor reporting is an impediment to appropriate implementation of research findings, then unwarranted or inappropriate use of research findings may expose patients to unnecessary risk of harm [5], while a failure to take up potentially beneficial research may lead to individual or population health benefits being missed [6]. This concept is important from a perspective of distributive justice, particularly if the failure to replicate research leads to an unwarranted burden on some members of society [7]. Being able to verify the veracity of the data and analysis will be essential in such circumstances. Moreover, there are opportunity costs to funding research. While clear reporting can facilitate replication and reproduction to validate findings, poorly reported research may precipitate a waste of resources if research funds are spent on unsuccessful attempts at reproduction that could have been circumvented had reporting been more complete. A number of studies have shown the difficulties in reproducing research findings [8, 9], with many citing poor reporting of methods for the inability to reproduce results.

### Reporting guidelines and meeting the ethical mandate

Reporting guidelines serve as a tool in supporting best practice in reporting. Indeed, several studies have indicated that the use of checklists improves the completeness of reporting [10–14]. The utility of reporting guidelines lies in clear reporting standards that not only serve as an aid for content in reporting, but may also prompt prospective consideration of pertinent issues during the conception of research [15].

Developed through a profession- or topic-specific consensus approach, reporting guidelines represent a form of self-regulation, as they become a set of *de facto* standards or professional norms. Their self-regulatory component reflects on the community’s desire to be seen as open, transparent, and honest – derived from the individual motivators for openness – yet guidelines also serve the external drivers; they are often endorsed, or compliance mandated, by journals [16]. By improving knowledge transfer, reporting guidelines may facilitate trustworthiness in, and accountability of, individuals under what O’Neill refers to as the ‘openness agenda’ and the ‘audit agenda’ [17]. Under the openness agenda, study reports that are compliant with best practice are used as a marker of trustworthiness and reflect the honesty of the author(s) through their willingness to transparently report their research. Under the audit agenda, transparent reporting improves accountability by facilitating inspection of performance with respect to professional standards [18]. More complete reporting – consistent with appropriate reporting guidelines – also assists the conduct of systematic reviews and meta-analyses through the better documentation of elements that would require extraction [18, 19]. Such improved reporting may increase the appropriate uptake of research findings.

## Conclusion

We have suggested that there are principled reasons behind the obligations to improve the reporting of research and note that this motivation reflects the integrity of individual researchers as well as the external drivers that pertain to the use of research results.

While reporting guidelines serve as a tool for multiple stakeholders, including researchers, prospective authors, and peer reviewers, questions remain regarding the extent to which they can influence processes beyond publication: to what extent do they influence researcher integrity or the uptake of scientific research findings into policy or practice? Put differently, how do they address the ethical drivers that have stimulated the production of reporting guidelines? Evaluations of reporting guidelines have tended to focus on intermediary outcomes or the extent to which published studies comply with the elements within guidelines. Even then, there has been surprisingly limited work in terms of guideline evaluation [20]. Indeed, the direct beneficiaries of reporting guidelines are generally researchers and those who peer review research [18].

We believe that the paucity of research on the efficacy and effectiveness of reporting guidelines is due to the complex nature of these interactions. It is difficult to attribute the endpoints, such as use of research in policy, to the way the study was reported. However, we believe that demonstrating the effectiveness of reporting guidelines is an important step in providing evidence of their applied benefits to both research funders and the broader public, as researchers seek to justify their work in an era of increasing financial constraints.

Hence, while improving the quality of reporting stems from important ethical drivers such as researcher integrity and the benefits derived from improved reporting, questions regarding the extent to which reporting guidelines can influence researcher integrity, or the uptake of scientific research findings into policy or practice, remain an important and under-investigated area for future research.
